# Health Risk Perceptions Are Associated with Domestic Use of Basic Water and Sanitation Services—Evidence from Rural Ethiopia

**DOI:** 10.3390/ijerph15102112

**Published:** 2018-09-26

**Authors:** Carmen Anthonj, Lisa Fleming, Samuel Godfrey, Argaw Ambelu, Jane Bevan, Ryan Cronk, Jamie Bartram

**Affiliations:** 1 Water Institute, Gillings School of Global Public Health, University of North Carolina, Chapel Hill, NC 27599, USA; lisaf@live.unc.edu (L.F.); rcronk@live.unc.edu (R.C.); jbartram@email.unc.edu (J.B.); 2 Water, Sanitation and Hygiene (WASH), UNICEF Ethiopia, P.O. Box 1169, Addis Ababa, Ethiopia; sgodfrey@unicef.org (S.G.); jbevan@unicef.org (J.B.); 3 Department of Environmental Health Sciences & Technology, Jimma University, P.O. Box 378, Jimma, Ethiopia; aambelu@yahoo.com

**Keywords:** behaviour change, diarrhoeal diseases, health knowledge, risk communication, health belief, rural water supply, SDG 6, WaSH intervention

## Abstract

We examine factors associated with the use of basic water supply and sanitation services as part of an integrated community-based nutrition programme which included a drinking water, sanitation and hygiene (WaSH) intervention and emphasise findings related to health risk perceptions. Data were collected from 2658 households in four regions in Ethiopia with a cross-sectional survey in WaSH intervention areas, as well as in control areas, where the intervention was not implemented. The data were analysed using bivariate and multivariable regression analysis. Awareness of health risk factors related to inadequate WaSH was high in the programme area. The use of basic water and sanitation services was associated with several health risk perceptions: Perceiving water quality as good increased the odds of using basic water services as opposed to believing the water quality was poor (OR 3.94; CI 3.06–5.08; *p* ≤ 0.001). Believing that drinking unsafe water was the main cause for diarrhoea increased the odds of using basic water services (OR 1.48; CI 1.20–1.81; *p* ≤ 0.001). In the WaSH intervention group, the use of basic sanitation was more likely than in the control group. The use of basic sanitation was associated with households who had previously received sanitation training, as opposed to such who had not (OR 1.55; CI 1.22–1.97; *p* ≤ 0.001). Perceiving dirty space as the main cause of diarrhoea (OR 1.81; CI 1.50–2.19; *p* ≤ 0.001), and privacy when using a latrine (OR 2.00; CI 1.67–2.40; *p* ≤ 0.001), were associated with higher odds of using basic sanitation. Households that indicated a disadvantage of owning a latrine was maintenance costs were less likely to use basic sanitation (OR 0.49; CI 0.38–0.63; *p* ≤ 0.001). Risk perceptions were important determinants of use of basic services. The findings point to risk perceptions motivating the application of positive WaSH-related and health-protective behaviours. This suggests that well-designed health risk communication strategies may be effective for engaging households in healthy WaSH behaviour.

## 1. Introduction

Evaluations of drinking water, sanitation and hygiene (WaSH) interventions in low- and middle-income countries (LMICs) facilitate improvement of global health and development policy making and implementation practice. WaSH interventions include the provision of new or improved water supplies, the improved distribution of water supplies by installation of hand pumps or household connection, the provision of water treatment for the removal of microbial contaminants at the source or at the point of water consumption, hygiene and health education and the encouragement of health-related behaviours, such as handwashing [1]. International organisations, academic institutions, governments, private actors and others have conducted studies to identify factors determining water service levels, household access to sanitation facilities [2], open defecation free (ODF) status [3], and handwashing with soap [4] to make WaSH interventions more effective on the long run.

### 1.1. Technical, Engineering, Sociological and Cultural Aspects and WaSH

While many studies examine the technical and engineering aspects of WaSH, such as functionality [5], water system breakdowns [6], access to sanitation [2], and sustainability of services [7], sociological, emotional distress and psychological aspects have received less attention [4,8,9,10]. However, as stated by Mara [11], rural water supply, sanitation and hygiene do not only incorporate engineering, but also sociology. Not only does ‘hardware’ play a role in determining WaSH services, but also the ‘software’: health risk perceptions, (mis)beliefs related to WaSH, WaSH-related behaviours, and the cultural context of WaSH [12,13,14]. These aspects are often neglected in project evaluations and studies on risk perceptions motivating WaSH-related behaviour are scarce.

### 1.2. Risk Perceptions as Motivators for Health- and WaSH-Related Behaviour

The perceptions of health risks refer to people’s intuitive judgments and evaluations of hazards they are or might be exposed to [15], the evaluation of which is influenced by a multitude of individual and societal factors. Such are based on experiences, beliefs, attitudes, judgements and feelings, as well as wider social, cultural and institutional processes [16]. Health-related knowledge strongly shapes the perceptions of risk.

Health-related knowledge and risk perceptions are motivators for the adoption of health-promoting WaSH behaviour [17,18]. This makes them useful to study, since access to safe, sufficient and continuously available drinking water, adequate sanitation and practice of appropriate hygiene behaviours form the foundation of human health and well-being, socio-economic development and human dignity [1,19,20,21,22,23,24]. However, health-related knowledge does not necessarily translate into healthy behavioural practice.

### 1.3. Achieving Safely Managed WaSH

Achieving safely managed water and sanitation for all is a priority in global development policy agendas. It is reflected in the United Nations General Assembly’s recognition of the human right to water and sanitation (Resolution 64/292) [25], as well as in the Sustainable Development Goals (SDGs) through Goal 6. Despite global commitments, many countries do not have universal access to safe WaSH services. In 2015, 2.1 billion people lacked access to safely managed drinking water services and 4.5 billion people lack safely managed sanitation services [26].

### 1.4. The Case of Rural Ethiopia

In Ethiopia, despite increases in water supply and sanitation coverage in rural areas and a favourable policy environment, there is considerable work to be done to achieve the National Growth and Transformation Plan II’s Water and Sanitation targets: 83% of the population using safely managed, adequate and resilient water supply services, and 100% of the population using safely managed and resilient sanitation services by 2020. The WHO/UNICEF Joint Monitoring Programme (JMP) estimates that 30% of the rural population had access to basic drinking water service in 2015, which includes drinking water from an improved source, with a collection time of less than 30 minutes for a roundtrip including queuing. As per definition of the JMP, improved drinking water sources are those which, by nature of their design and construction, have the potential to deliver safe water, and include: piped water, boreholes or tubewells, protected dug wells, protected springs, rainwater, and packaged or delivered water. As few as four percent of the population use basic sanitation, defined as an improved facility which is not shared with other households. Improved sanitation facilities are those designed to hygienically separate excreta from human contact, and include: flush/pour flush to piped sewer system, septic tanks or pit latrines; ventilated improved pit latrines, composting toilets or pit latrines with slabs [27].

More than 80 percent of the Ethiopian population relies on agriculture and livestock for their livelihoods, and these have been undermined by droughts [28]. As a result, about 8.3 million Ethiopians—ten percent of the population—remain food insecure due to low agricultural productivity at a household level.

Increasing water scarcity, brought about by extreme climatic conditions, as well as environmental degradation may compound inadequate access to WaSH services, while also increasing food and nutrition insecurity and malnutrition among affected populations even further [29].

This study from rural Ethiopia aimed at (i) identifying WaSH-related factors and practices, socioeconomic and behavioural aspects, as well as risk perceptions and health beliefs associated with the domestic use of basic drinking water and sanitation service levels; and at (ii) exploring differences between the WaSH intervention group and control group two years after the completion of an integrated nutrition and WaSH programme.

## 2. Materials and Methods

A cross-sectional household survey was conducted in Amhara, Oromia, Tigray and Southern Nations, Nationalities, and Peoples’ Region (SSNRP) in Ethiopia. Data were collected between January and March 2017 by the Water Institute at UNC and Jimma University on behalf of UNICEF Ethiopia.

### 2.1. Study Context

In the context of food insecurity and malnutrition, and in order to maximise the potential health impact of community-based nutrition (CBN) programmes among vulnerable groups, an integrated nutrition and WaSH programme was implemented by UNICEF Ethiopia and the Government of Ethiopia between 2011 and 2015. It was designed to respond to the combined risks of chronic malnutrition and inadequate access to basic WaSH services for 1.4 million people in four regions in rural Ethiopia. Part of the project was an investigation of use-related behaviours regarding water and sanitation services.

Thirty intervention woredas (districts) containing 576 kebeles (communities) were subject to the large-scale WaSH intervention. Rural water supply (CWS) was provided through 1800 community managed water supply systems benefiting a population of 630,000 people. Water schemes were constructed through community-managed and self-supply approaches. A focus was set on multiple use services (MUS). A community-led total sanitation and hygiene promotion (CH&S) component resulting in home-built toilets benefited an estimated 280,000 families. A capacity and knowledge dissemination component for behaviour and social change, through manuals, guidelines and research in the intervention communities was part of the programme as well.

**Figure 1:**
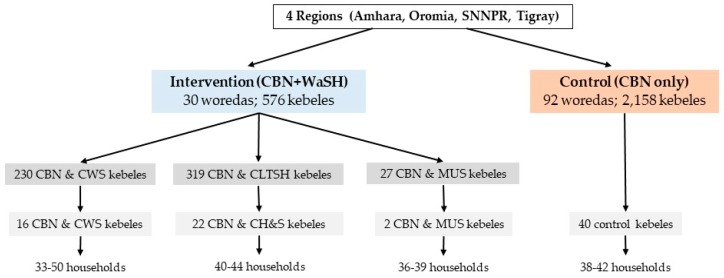
Sampling methodology.

The control areas, which contained 92 woredas and 2158 kebeles, were targeted with CBN programming (Figure 1).

### 2.2. Sampling

Eighty representative kebeles (40 intervention and 40 control) were selected and out of these kebeles, one village was selected at random. The number of households surveyed within each kebele was determined using the proportional distributions for each intervention group. Households were chosen systematically in a village based on the World Health Organization Expanded Programme on Immunisation (EPI) method. All households within each selected kebele were eligible to be interviewed. 1221 households were sampled in the intervention groups and 1437 in the control group resulting in a total of 2658 households.

### 2.3. Data Collection Based on Structured Surveys

Data collection was carried out by 21 field enumerators and four supervisors recruited by Jimma University and trained by Jimma University and the Water Institute at UNC. The enumerators were recruited from study areas, mostly had an education in disciplines related to the environment or health and all were experienced in data collection. Structured surveys addressed general household information and questions related to the drinking water source (e.g., type of water point, distance and time to collect water, amount of water collected, cost, perceived water quality), storage and treatment, sanitation (type of facility, location, sharing of facility, cleaning and emptying, training/sensitisation) and handwashing, as well as health- and WaSH-related knowledge and risk perceptions (factors increasing and preventing diarrhoea risk, benefits and disadvantages of latrines). Additionally, observational water source and sanitation spot checks were carried out. The surveys were conducted in teams of two enumerators in the most appropriate of the three most prominent local languages (Amharic, Oromifa and Tigrigna). The data were collected using Android smartphones with the pre-installed SurveyCTO mobile data platform.

Ethical clearance was obtained both from the Institutional Review Board of the College of Health Science at Jimma University, Ethiopia (RPGC/967/2016) and from the University of North Carolina at Chapel Hill (study #15-3317).

### 2.4. Data Analysis

The three different WaSH intervention groups (CWS, CH&S, MUS) were merged into a combined CBN + WaSH arm for analysis, which was compared to the control group, where only CBN was implemented.

Descriptive statistics were calculated and included frequencies for all variables of interest, as well as means and/or quintiles for numerical variables.

Bivariate regression analyses were conducted to examine the strength of association between the predictor variables (independent variables; binary coded) and the primary outcome variables, namely the availability of basic water and basic sanitation services at the household level (dependent variables, separate models were run for basic water and for basic sanitation). The outcome variables for basic water and sanitation services were created by merging several variables, related to drinking water source type, time to fetch water for basic water service, sanitation facility, and sharing of facilities with other households for basic sanitation (UNICEF & WHO 2015). A 95% confidence interval (CI) was used to estimate the precision of the odds ratios (OR) (significance level set at *p*-value ≤ 0.05).

Multivariable models were generated to examine the factors that predict the household use of basic water and sanitation services. All analysis was conducted using STATA 15 (StataCorp LLC, College Station, TX, USA).

## 3. Results

The households surveyed in intervention (CBN + WaSH) and control group (CBN only) areas had similar characteristics (Table 1). About half of the households had between four and six people, and 91% were headed by men. Most heads of households were farmers (90% of CBN + WaSH areas; 82% of CBN areas).

More than half of the household heads and about two thirds of the mothers or caregivers lacked a formal education. Less than one percent had higher education beyond secondary level or vocational training. Electricity, used as a proxy for wealth, was available in 20% of the intervention and in 25% of the control households.

### 3.1. Water, Sanitation and Risk Perceptions

#### 3.1.1. Water Source, Storage, Treatment and Access

**Figure 2:**
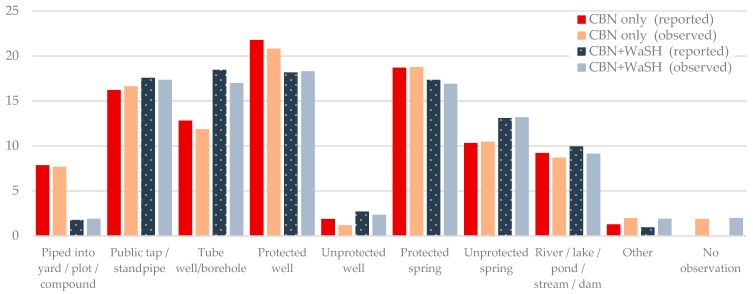
Main drinking water source (reported and observed) (%).

The main drinking water sources used by the households were protected wells, protected springs, public taps and standpipes. Water piped into dwellings, tanker trucks or harvested rainwater were rarely used. The observed water sources differed from the sources reported by the households (Figure 2).

More than three quarters of all households used improved drinking water sources (68% in CBN + WaSH; 74% in CBN). Adult women were the primary collectors of water (79% in CBN + WaSH; 76% in CBN) and most commonly used jerry cans (more than 90%). Drinking water was stored in the household primarily in a container with a lid (98%).

**Figure 3:**
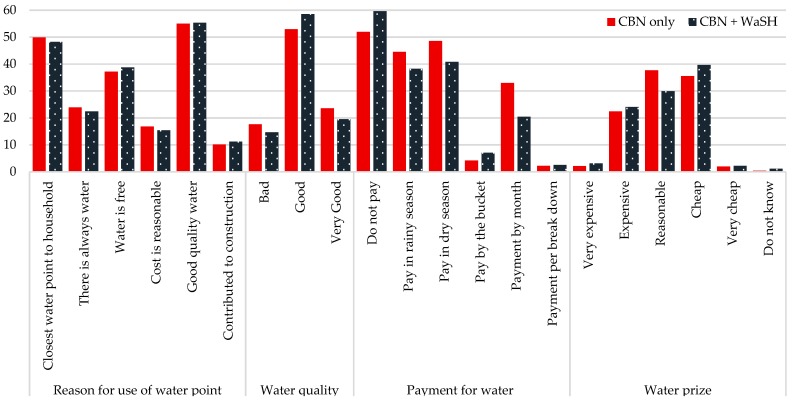
Use of water point, water quality and cost related to water (%).

The households collected water from those sources that were closest to their households (59%), that they believed had good water quality (55%), and where water was free of charge (38%). More than two thirds of households perceived the water from their primary source to be of good quality. Many of those who reported paying for their water (45%) described the drinking water cheap (37%) or reasonably priced (34%) (Figure 3). In the month preceding the survey, households paid, on average, 24 KSH (~0.24 USD) to cover water-related costs.

Distance to the primary water source, time needed to reach the water point, and volume of water collected was similar in the intervention and control areas. About half of all households travel more than half a kilometer to their primary water source.

**Figure 4:**
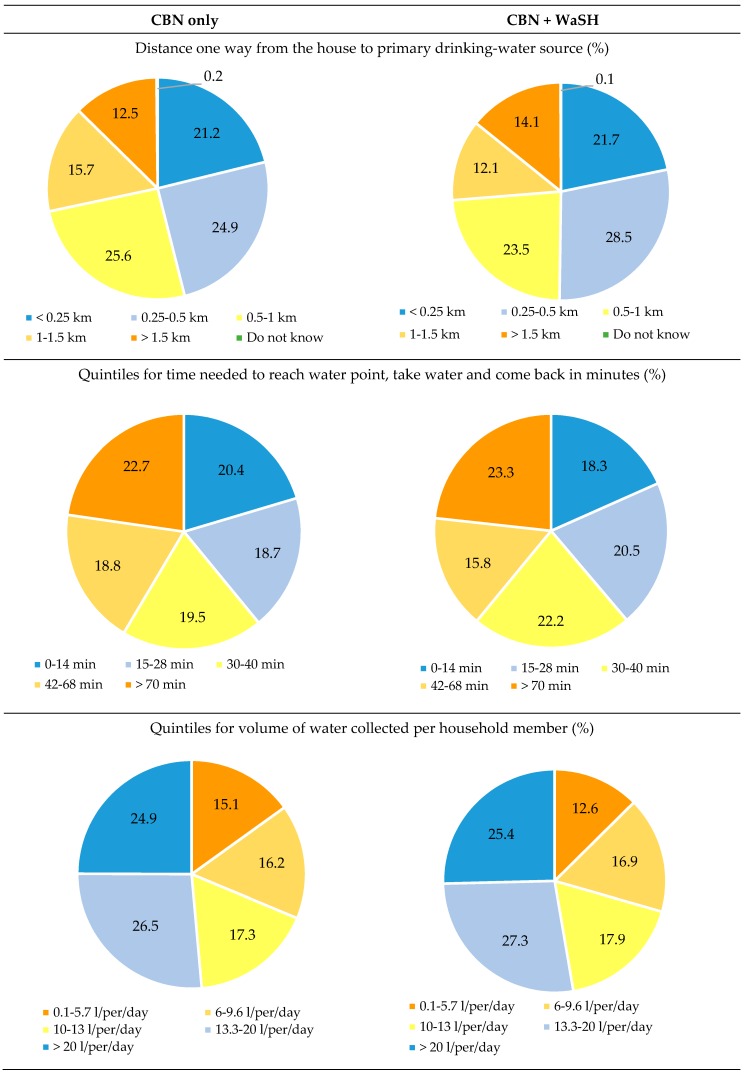
Water access: distance and time needed to access water point, amount of water collected.

About 60% of households took more than 30 minutes roundtrip when collecting water. About 30% of the households collected less than 10 liter per person per day per household member (Figure 4).

Most households used the drinking water source for multiple purposes; mainly for cooking (90% in CBN + WaSH; 94% in CBN), handwashing (88% in CBN + WaSH; 92% in CBN), and bathing (73% in CBN + WaSH; 79% in CBN). 9% of households in the intervention area and 5% in the control area used the primary source only for drinking, and 2% used the water for irrigating their fields.

#### 3.1.2. Sanitation Type, Location and Latest Sanitation Training

**Figure 5:**
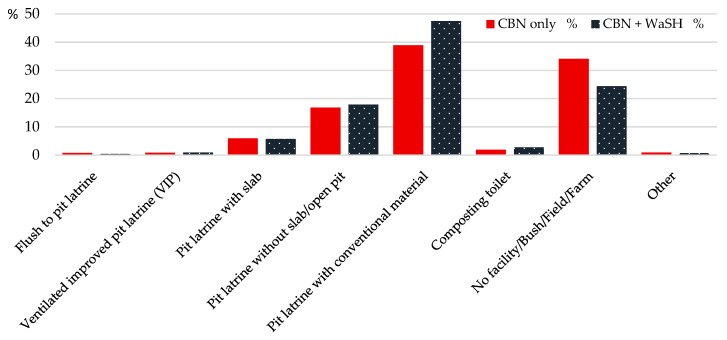
Observed sanitation facilities in communities.

The sanitation facilities that were mostly observed included pit latrines constructed with conventional materials (trees, bamboo, tins) (47% in CBN + WaSH; 39% in CBN), pit latrines without slabs and with open pits (17%) and pit latrines with slabs (6%). According to the household heads interviewed, more than half of all household members (58%) used improved sanitation. Unimproved sanitation was used by about 18% of the household members, and by almost one third of school-aged children. The households in the intervention areas reported a higher level of use of improved sanitation. In 24% of households in the intervention areas and in 34% of households in the control areas, no sanitation facility was observed (Figure 5).

Most facilities were reported to be located on the compound (89% in both groups), few shared their facilities with other households (9% in CBN + WaSH; 12% in CBN). Nearly all facilities were observed to be functional (98% in CBN + WaSH; 96% in CBN).

Most households’ latrines were observed to be in a good condition in terms of their structure (85%), and were reported to have been emptied at least once (71%). Although most households claimed to clean the facilities when needed (71% in CBN + WaSH; 66% in CBN), only 19% of those were observed to be clean (Table 2).

Almost 80% of all respondents had taken part in sanitation trainings or sensitisation programmes before. For the month preceding the survey, the intervention group reported higher participation in such programmes (28% in CBN + WaSH; 17% in CBN). In both groups, 45% of the households had taken part in such activities within the past year. In the intervention group, 15% had never participated in any sanitation programme, and 22% of the members of the control group never participated in a sanitation programme.

#### 3.1.3. Risk Perceptions Related to Sanitation and Diarrhoea

The household heads believed that sanitation facilities provided benefits as well as disadvantages (Table 3). The presence of a latrine was perceived to contribute to the cleanliness of the compound and to better health by more than 80% of the households, while fewer saw the contribution of latrines to privacy, safety and social status as beneficial. Drawbacks of latrines included costs of construction (30%) and maintenance (17%), and bad smell and pollution of the compound (9%). The intervention group saw more positive and less negative aspects as compared to the control group.

Most respondents believed that the presence of human faeces (93%) and flies in contact with faeces via food (96%) caused diarrhoea, as well as animal faeces on the compound (69%). The main measures perceived to prevent diarrhoea were washing hands with soap (90%) or ash (64%). Fewer respondents believed that washing with water only (27%) or only once a day (54%) were sufficient. The most common health-promoting measures to prevent diarrhoea included good hygiene practices (74%), washing hands before eating (71%), household cleanliness (66%), and drinking safe water (56%). The least reported included the use of latrines (24%) and water treatment (14%).

### 3.2. Factors Associated with the Use of a Basic Water Service

#### 3.2.1. Findings from Bivariate Regression Analyses

In bivariate analyses, the use of a basic water service by households was associated with the region, the presence of electricity, the highest level of school education, reasons for using the water point such as proximity, availability, cost, quality and ownership, payment for water and with various WaSH-related health (mis)beliefs regarding advantages and disadvantages of the use of latrines, as well as causes and preventive measures for diarrhoeal diseases (Table 4).

Households in Tigray were less likely to use basic water services as compared to households in Amhara (OR 0.79; CI 0.63–1.03; *p* = 0.035). Households with electricity had increased odds of basic water services in the unadjusted model, but reduced odds in the adjusted model (OR 0.80; CI 0.66–0.96; *p* = 0.016). The likelihood of using a basic water service was positively associated with the highest level of formal education of the household head (OR 1.07; CI 1.02–1.13; *p* = 0.004).

Multiple use of water points besides drinking significantly decreased the odds of using a basic service in the unadjusted model, but increased the odds in the adjusted model as compared to using the source for drinking only (OR 1.77; CI 141–2.21; *p* ≤ 0.001). Using the source because the water was free significantly reduced the odds (OR 0.85; CI 0.72–1.00; *p* = 0.046) of the source qualifying as a basic water service.

Payment for water was associated with the availability of a basic water service. Household heads that paid by bucket, for example, had higher odds (OR 1.70; CI 1.18–2.43; *p* = 0.004) of using basic water services than those households that did not. Paying for water by month (OR 0.58; CI 0.49–0.70, *p* ≤ 0.001) or paying for water in the rainy and dry season (OR 0.73; CI 1.20–1.48; *p* ≤ 0.001) significantly reduced the odds of use of a basic water service.

Believing that a dirty household environment (OR 1.20; CI 1.03–1.41; *p* = 0.024) or poor food hygiene (OR 1.24; CI 1.04–1.48; *p* = 0.016) were main causes of diarrhoea were significantly associated with the use of basic water service.

#### 3.2.2. Findings from Multivariable Regression Analyses

The results of the multivariable model suggest household heads with electricity are more likely to use a basic water service (OR 2.45; CI 1.90–3.01; *p* ≤ 0.001) as compared households that had none (Table 5).

Multiple uses of the water point besides drinking significantly reduced the households’ odds of using a basic water service (OR 0.63; CI 0.48–0.84; *p* = 0.001). Perceiving the water quality as good (OR 3.94; CI 3.06–5.08; *p* ≤ 0.001) and believing that drinking unsafe water was the main cause for diarrhoea (OR 1.48; CI 1.20–1.81; *p* ≤ 0.001) significantly reduced the odds.

### 3.3. Factors Associated with the Use of a Basic Sanitation Service

#### 3.3.1. Findings from Bivariate Regression Analyses

In bivariate analyses, the use of basic sanitation was associated with electricity, sanitary hygiene, sanitation training and sensitisation activities and with risk perceptions in bivariate analyses (Table 6). Household electricity significantly increased the odds (OR 1.30; CI 1.04–1.63; *p* = 0.022).

Households with their latrine structure observed to be in good condition were significantly more likely to use basic sanitation services (OR 1.31; CI 1.12–1.53; *p* < 0.001) as compared to those that did not. Household heads having received a sanitation training had significantly increased odds of using basic sanitation services in the unadjusted model, but significantly reduced odds in the adjusted model.

Perceptions on benefits and disadvantages of latrines, as well as on main reasons for diarrhoea were associated with the use of a basic sanitation service. Those household heads believing that a latrine would improve health had significantly higher odds (OR 1.23; CI 1.03–1.47; *p* = 0.020) of using basic sanitation services. Respondents who believed that their latrine provided privacy had significantly increased odds of using a basic sanitation service in the unadjusted model, but significantly reduced the odds in the adjusted model. Household heads believing disadvantages of latrines included an increased diarrhoea risk (OR 1.20; CI 1.03–1.41; *p* = 0.024) had higher odds of using basic sanitation services. A reduced likelihood of basic sanitation service use was calculated for household heads who believed that the construction (OR 0.78; CI 0.64–0.96; *p* = 0.020) of latrines was very costly. Believing that drinking unsafe water (OR 1.30, CI 1.04–1.63; *p* = 0.022) was the main cause for diarrhoea significantly increased the odds of using basic sanitation service as compared to those who did not.

#### 3.3.2. Findings from Multivariable Regression Analyses

The results of the multivariable model reveal that the household heads of the intervention group (CBN + WaSH) were significantly more likely to use basic sanitation (OR 1.41; CI 1.18–1.69; *p* ≤ 0.001) than the control group (Table 7). The model suggests the use of basic sanitation services to be associated with the region, e.g., household heads in SNNPR using basic services more than in Amhara (OR 1.58; CI 1.26–1.99; *p* ≤ 0.001).

The odds of using a basic sanitation service was significantly increased by regular maintenance of a facility such as having had the latrine emptied at least once in the past (OR 6.00; CI 4.86–7.40; *p* = < 0.001), and so did having received sanitation training (OR 1.55; CI 1.22–1.97; *p* ≤ 0.001).

Believing that a dirty space was the main cause for diarrhoea significantly increased the odds of a household head using basic sanitation service (OR 1.81; CI 1.50–2.19; *p* ≤ 0.001), and so did considering the improved privacy due to a latrine (OR 2.00; CI 1.67–2.40; *p* ≤ 0.001). Household heads that indicated that a disadvantage of owning a latrine was the cost of its maintenance were significantly less likely to use basic sanitation service (OR 0.49; CI 0.38–0.63; *p* ≤ 0.001).

## 4. Discussion

We describe the status of and factors associated with the use of basic water and sanitation services based on data from a combined WaSH and nutrition intervention programme in Ethiopia. Region, intervention and control area, socioeconomic status, multiple use of the main water source, sanitary hygiene, sanitation sensitisation and training, educational background and health risk perceptions determined the use of basic services.

### 4.1. Factors Associated with Basic Water and Sanitation Services

The use of basic sanitation services was associated with the region (SNNPR had higher odds than Amhara), while basic water services was not. The four regions where the community-based nutrition programme was implemented are very diverse in terms of environment, topography, hydrology, regional climate and occurrence of extreme weather events, and they are inhabited by different ethnic, cultural and linguistic population groups that settle in different population densities and have different lifestyles, all of which are factors that may explain these differences [2,12,13].

Households that had electricity were positively associated with basic water services as compared to households who lacked electricity. No respective association was found regarding the use of basic sanitation services. In this study, the presence of electricity was used as a proxy for socioeconomic status. Consequently, the interpretation of these results would point to a better socioeconomic status increasing the use of basic water services, but not of basic sanitation services. While the presence of electricity surely points to a better socioeconomic situation as compared to households who lack electricity, the findings may suggest that electricity alone is not an entirely robust indicator. In a rural setting, as in the programme area, electricity may be present in certain villages and absent in others; thus, it is a rather weak indicator for wealth as compared to wealth indices that, in addition to electricity, include the possession of other additional asset items [30,31].

Household heads who used their main drinking water source for multiple purposes other than drinking (including cooking, hygiene, washing) were less likely to use basic services as compared to those only using the main drinking water source for drinking only. This may suggest that households who drew their combined water needs from just only one source were forced to use an unimproved service, possibly because the basic services could not produce the quantities needed to address all water needs in the water-scarce rural area. This finding underlines the need for actors and researchers to not only focus on, but also encourage the use of multiple water sources for different purposes [32].

Households whose latrine had been emptied at least once before were more likely to use basic sanitation services that were not shared with others. This underlines that besides presence, sanitary hygiene matters in terms of use.

### 4.2. Differences in Basic Services between the Intervention and Control Areas

In the programme area, where 86% of the households rely on agriculture as their livelihoods, but where chronic malnutrition is widespread, the use of basic services is vitally important. Ensuring that sufficient water is available not only for WaSH and domestic purposes, but also for irrigation has a substantial impact on food availability; it improves nutritional status, and thus human health. The use of unsafely managed water supply (for both domestic and local productive use), inadequate access to sanitation, and poor hygiene practices, however, exacerbates food insecurity and malnutrition, while at the same time decreasing work productivity further.

This programme evaluation revealed no significant difference in the use of basic water services between intervention and control areas, which suggests that the households in the control areas perform as well as do the households in the intervention area.

The use of basic sanitation services was significantly higher in intervention than in control areas. Moreover, the respondents from the intervention area were more likely to link latrine use to health benefits, which suggests that sanitation sensitisation in the intervention may have been successful.

The data of this programme implementation was collected two years after completion of the WaSH intervention. Thus, assuming a positive effect on WaSH behaviour and on WaSH-related knowledge and risk perceptions as entirely owing to the intervention may not suffice, as a substantial amount of time has passed since.

### 4.3. The Implications of Education on Basic Water and Sanitation Services

Education levels were low in the programme area; about 51% of the household heads never went to school, and 8% received only informal or pre-school education. Thus, only about 40% ever went to primary or higher schools. Households with heads who had received any education were significantly more likely to use basic water services as opposed to those who received no education at all, as shown in the bivariate analyses. Education did not show any significant association with the use of basic sanitation services. The household head’s education was not significantly associated with either the use of basic water or sanitation services in the multivariable models. Thus, formal education plays a limited role in terms for the use of basic services, contradicting studies from similar settings [2,13,33].

In the programme area, education was measured by years spent at school. However, knowledge can be acquired elsewhere, e.g., via the radio, newspapers or internet broadcast, at health centres, through community health workers and family members, and is not necessarily measurable or quantifiable.

The fact that household heads in the programme area who had received sanitation sensitisation or training before were more likely to use basic sanitation service than those who had not, underlines this statement, and indicates that the education component of the intervention may have been successful. This becomes visible due to the high level of risk knowledge and perception related to diarrhoea in the programme area, the low overall education level notwithstanding. These findings also acknowledge the importance of strengthening all health educators, i.e., community health workers’ role in the implementation of WaSH [34,35].

### 4.4. The Role of Risk Perceptions for WaSH-Related Behaviour

Despite the low level of formal education, the awareness on risk factors related to WaSH and diarrhoea was high in the programme area. The disease was believed to be linked to faeces, the presence of flies, poor food hygiene, ‘dirty spaces’ and unsafe drinking water.

The household members’ perceptions adequately reflect the real WaSH-related risks, as described in the framework on faecal-oral disease transmission (‘F-diagram’) on transmission routes of excreta-related pathogens that cause disease [36,37], thus supporting evidence from Kenya, where the risk perceptions from the grassroots level corresponded to real health risks [34,38].

The study from Ethiopia may go one step beyond: In this study, household heads attributed numerous benefits, such as better health or cleanliness of the compound, and disadvantages, such as construction and maintenance cost, to the use of latrines. Believing that unsafe drinking water was the main cause for diarrhoea, for example, proved to be positively associated with the use of basic water service (OR 1.48; CI 1.20–1.81; *p* ≤ 0.001). The perception of ‘dirty spaces’ causing diarrhoea was associated with the use of basic sanitation service (OR 1.81; CI 1.50–2.19; *p* ≤ 0.001). Moreover, household heads perceiving the water quality of the main drinking water source as good was positively associated with the use of basic water service as opposed to those perceiving the water quality to be poor (OR 3.94; CI 3.06–5.08; *p* ≤ 0.001). Believing that the quality was good therefore animated the household heads to make use of a particular water point. While this does not prove that the water quality free of contamination based on microbial or chemical water testing, it indicates that health risk perceptions matter. Both findings point to risk perceptions being closely linked to and potentially motivating the application of positive WaSH-related and health-protective behaviour—the use of basic services. These results confirm findings from a qualitative study in Malawi that found risk communication on the need for domestic water treatment effecting behaviour change [39].

Although the benefits of safely managed sanitation were clear to the respondents, only about 20% of the observed sanitation facilities were clean, and about 30% had never emptied. This could mean that many of the latrines were relatively new and had therefore never been emptied. This could also mean, as is common in some rural areas, that latrines were not emptied, but topped off, and the slab and infrastructure moved to a new pit. So, do risk perception and health beliefs thus not translate into practice?

Some household heads’ risk perceptions were positively or negatively associated with the use of basic sanitation service. Those, for example, who believed that the main benefits of latrines included improved privacy were more likely to use a basic sanitation service, whereas those who believed that the main disadvantage of a latrine was its maintenance cost were less likely to use basic sanitation services. Thus, some of the risk perceptions are put in practice, while others are not.

Although risk perceptions may act as triggers for precautionary action [40], it should be noted that the engagement in preventive health behaviours is not merely determined by the awareness of objective health risks, but is also greatly influenced by health beliefs and specific health cognitions [41]. Thus, risk perceptions do not necessarily translate into practice and the engagement in preventive health behaviours [17,18]. Practising healthy behaviour is dependent upon a variety of social, cultural and economic factors, and not limited to infrastructure development and education [13,41,42]. According to Curtis et al. [17,18] and following social and evolutionary psychology and neuroscience, health and WaSH-related behaviour can be assigned to three types of interacting causes. These are cognitive or executive control producing ‘planned’ behaviour, aimed at preventing disease, achieving long-term health goals, and adequate socialisation. Moreover, there is the reward system stimulating ‘motivated’ behaviour, with drivers of motivation being disgust, status and social standing, and attraction, as well as fear of WaSH-related diseases. Additionally, there is the automatic or reflexive control, which is responsible for ‘habitual’ behaviour, learnt at an early age, automated and regularly triggered by a particular cue. Considering that 80% of respondents had taken part in sanitation training or sensitisation activity, which was positively associated with use of basic sanitation service, and given that risk perception is a major motivator for behaviour change [43], well-designed communication strategies and health messaging could speak to a highly effective form to engage households to accept and use basic services [33,39,44].

### 4.5. Limitations

A limitation lies in the cross-sectional design of the survey, which was useful for providing a snapshot of the situation and for associating the use of basic water and sanitation services with different explanatory factors, but, however, could not account for behaviours at different points of time. Originally, this study was supposed to compare not only the intervention and control areas, but also baseline data with post-intervention data. Due to major quality issues with the baseline data, no proper comparison was possible. Such would have allowed uncovering cause-effect relationships, e.g., in terms of seasonal differences in terms of use of services [45].

Qualitative data, collected through open-ended questions as part of the household survey, in-depth interviews and focus group discussions, or through anthropological techniques such as photovoice [14], would have added vital value to the findings of this study. Particularly in terms of health risk perceptions [34] related to WaSH, which can only partly be captured by quantitative research, such approaches should be included in future studies for the sake of triangulation of findings [12,13]. Risk perceptions and health beliefs are complex, multi-dimensional and influenced by cultural practices and social factors [2,14,33,46,47]. They can motivate the application of positive WaSH-related choices and health-related behaviours, inform health-related management [34] and should therefore play a more prominent role in the design of WaSH programmes and evaluations.

This study included households that were mainly headed by males (91%). The small number of female-headed households detected prevented sex-disaggregation. The burden of limited or unimproved water and sanitation services, however, falls disproportionately on women, who bear responsibility for all related domestic tasks [39], while caring for the children and the sick. Thus, viewing WaSH in the programme areas through a gender lens by comparing male- with female-headed households may have uncovered differences in terms of risk perceptions, as well as additional explanatory factors to the use of basic services.

## 5. Conclusions

This study identified regional variations and differences between intervention and control areas, socioeconomic status, multiple use of the main water source, sanitation sensitisation/training, and educational background to be factors significantly associated with the use of basic water and sanitation services in a community-based nutrition programme area in four Ethiopian regions. This supports evidence from previous studies.

The novelty of this study lies in the role of health risk perceptions as important determinants of the domestic use of basic water and sanitation services, thus pointing to risk perceptions motivating the application of positive WaSH-related and health-protective behaviours. Previous studies have discussed health-related knowledge and level of formal education as being crucial in determining behaviour [17,18,43]. However, investigations of health risk perceptions were scarce, and the gap of risk perceptions actually translating into practice was criticised.

This study underlines the need to close the perception to action gap. It demonstrates the importance of integrating community risk perceptions in risk communication strategies and health messaging to constitute a highly effective form to engage households to accept and use basic services. With their potential to motivate households to ‘climb up’ the WaSH service ladders, risk perceptions are ultimately relevant for the achievement of the SDG 6 [26].

## Figures and Tables

**Table 1:** Characterisation of households in intervention and control groups.

Characteristics	CBN Only		CBN + WaSH	
	*N*	(%)	*N*	(%)
Region				
Amhara	397	32.51	501	34.86
Oromia	142	11.63	211	14.68
SNNPR	465	38.08	507	35.28
Tigray	217	17.77	218	15.17
Information on household				
Household head (male)	944	90.86	1130	91.35
1–3 people	330	27.03	386	26.86
4 people	192	15.72	220	15.31
5 people	209	17.12	249	17.33
6 people	201	16.46	227	15.80
7–14 people	289	23.67	355	24.70
Electricity available	288	24.57	274	20.06
Occupation of household head				
Farming	852	82.00	1109	89.80
Business/Traders	61	5.87	36	2.91
Permanent wage work	44	4.23	23	1.86
Self-employed	17	1.64	3	0.24
Daily laborer	16	1.54	10	0.81
Retired/old age	16	1.54	17	1.38
Other	33	3.18	37	2.99
Highest education level completed by household head				
No school	539	51.93	621	50.28
Informal or pre-school	61	5.88	130	10.52
Primary (1–6)	239	23.03	308	24.94
Secondary (7–12)	159	15.32	161	13.04
Technical/Vocational	10	0.96	4	0.32
Higher	30	2.89	11	0.89
Highest education level completed by mother/caregiver				
No school	627	65.45	770	67.31
Informal or pre-school	20	2.09	35	3.05
Primary (class 1–6)	201	20.98	242	21.15
Secondary (class 7–12)	91	9.50	95	8.30
Technical/Vocational	10	1.04	1	0.09
Higher level beyond the above	9	0.94	1	0.09

**Table 2:** Sanitary conditions of the sanitation facility.

Sanitation Facility: Hygiene and Structure	CBN Only		CBN + WaSH	
	*N*	%	*N*	%
Only cleaned when needed	526	65.83	748	70.77
Emptied at least once	920	72.27	1050	70.71
Never emptied before	353	27.73	435	29.29
Currently observed to be clean	162	20.56	187	17.54
Structure currently observed to be in good condition	664	84.26	918	86.12

**Table 3:** Health risk perceptions related to sanitation and diarrhea.

Risk Perceptions and Behaviours	CBN Only		CBN + WaSH	
	*N*	%	*N*	%
*Opinion of main benefits of latrine*				
Clean compound	1029	85.11	1236	88.16
Better health	991	81.97	1191	84.95
Easier/safer at night	292	24.15	395	28.17
Better privacy	489	40.45	602	42.94
Better social position	240	19.85	327	23.32
*Opinion of main disadvantage of latrine*				
Construction costs	394	32.59	391	27.89
Maintenance costs	227	18.78	215	15.34
Cleaning work	48	3.97	68	4.85
Dark	24	1.99	48	3.42
Small space	108	8.93	124	8.84
Bad smell, dirt	230	19.02	257	18.33
Reason for diarrhoea	82	6.78	120	8.56
*Perceived reasons for diarrhoea*				
Human faeces	1079	92.06	1278	93.56
Presence of animal faeces in compound	790	67.41	965	70.64
Flies in contact with faeces via food	1108	94.54	1325	97
Mosquitos	607	51.79	698	51.1
*Perceived measures that prevent diarrhoea*				
Washing hands with water only	334	28.5	366	26.79
Washing hands with ash	733	62.54	906	66.33
Washing hands with soap	1047	89.33	1241	90.85
Washing hands once a day is enough	193	16.47	174	12.74
*Activities at the household to prevent diseases, especially diarrhoea*				
Drink safe water	649	55.38	793	58.05
Water treatment	155	13.23	194	14.2
Use of the latrine	277	23.63	334	24.45
Good hygiene practices	846	72.18	1039	76.06
Wash hands after using latrine	633	54.01	795	58.2
Wash hands before eating	799	68.17	1011	74.01
Covering the food	660	56.31	855	62.59
Household cleanliness	727	62.03	946	69.25

**Table 4:** Bivariate logistic regression results for the use of basic water services.

Outcome is Use of Basic Water Service	Unadjusted Model				Adjusted Model			
		CI 95%				CI 95%		
	OR	Low	Up	*p*-Value	OR	Low	Up	*p*-Value
Explanatory variable								
*Region*				Interv vs. contr	0.90	0.77	1.05	0.192
Oromia vs. Amhara	1.05	0.82	1.35	0.696	1.24	0.97	1.59	0.084
SNNPR vs. Amhara	0.97	0.80	1.16	0.715	0.86	0.72	1.03	0.112
Tigray vs. Amhara	1.59	1.26	1.99	**<0.001**	0.79	0.63	0.98	**0.035**
Household has electricity * vs. none	3.00	2.48	3.63	**<0.001**	0.80	0.66	0.96	**0.016**
Household head’s highest level of formal education	1.08	1.03	1.13	**0.003**	1.07	1.02	1.13	**0.004**
Mother’s highest level of formal education	1.05	0.99	1.11	0.102	1.05	0.99	1.10	0.116
MUS of water point vs. no	0.72	0.58	0.91	0.006	1.77	1.41	2.21	**<0.001**
*Reason for using this water point*								
Closest water point to household vs. no	1.55	1.33	1.82	**<0.001**	0.94	0.80	1.09	0.398
Short waiting time vs. long	1.52	1.27	1.83	**<0.001**	0.92	0.77	1.10	0.362
Water is always available vs. no	1.45	1.23	1.70	**<0.001**	1.07	0.91	1.25	0.403
Water is free vs. no	0.79	0.67	0.94	**0.006**	0.85	0.72	1.00	**0.046**
Cost for water is reasonable vs. no	2.27	1.83	2.80	**<0.001**	0.90	0.73	1.11	0.337
Water quality is good vs. not good	3.49	2.94	4.13	**<0.001**	1.01	0.87	1.18	0.888
Household contributed to construction vs. no	1.89	1.48	2.43	**<0.001**	1.12	0.87	1.44	0.361
*Payment for water*								
Paid money in the last month vs. no	0.51	0.40	0.64	**<0.001**	1.05	0.85	1.30	0.633
Water price is expensive vs. not expensive	1.23	0.95	1.60	0.123	0.79	0.60	1.02	0.072
Paid for water last time vs. no	0.85	0.65	1.11	0.233	1.12	0.86	1.46	0.403
Household paid for water in dry season vs. no	2.07	1.76	2.42	**<0.001**	0.73	0.63	0.85	**<0.001**
No payment vs. payment	0.46	0.39	0.54	**<0.001**	1.28	1.09	1.50	**0.002**
By bucket vs. other	1.93	1.37	2.74	**<0.001**	1.70	1.18	2.43	**0.004**
By month vs. other	2.09	1.75	2.51	**<0.001**	0.58	0.49	0.70	**<0.001**
Per breakdown vs. other	1.19	0.64	2.22	0.581	1.68	0.90	3.15	0.105
Never paid/water point never broke vs. yes	0.74	0.62	0.88	**0.001**	0.81	0.68	0.96	**0.014**
Perceived water quality as good vs. no	4.52	3.63	5.62	**<0.001**	0.72	0.60	0.86	**<0.001**
Household paid for water in rainy season vs. no	2.16	1.84	2.53	**<0.001**	0.78	0.67	0.91	**0.002**
Household paid for water in any season vs. no	2.07	1.76	2.42	**<0.001**	0.73	1.20	1.48	**<0.001**
*Health risk perceptions: main reason for diarrhoea*								
Drinking unsafe water vs. no	1.44	1.22	1.71	**<0.001**	1.10	0.93	1.29	0.269
Dirty space vs. no	1.35	1.14	1.59	**<0.001**	1.20	1.03	1.41	**0.024**
Not washing hand with soap vs. yes	1.20	1.02	1.40	**0.028**	1.16	0.99	1.35	0.065
Defecate in the open air vs. no	1.31	1.12	1.53	**0.001**	1.12	0.96	1.31	0.152
Poor food hygiene vs. no	1.41	1.17	1.69	**<0.001**	1.24	1.04	1.48	**0.016**
Parasites in the faeces vs. no	1.62	1.33	1.98	**<0.001**	1.13	0.92	1.38	0.243

* Electricity used as a proxy variable to wealth in this study. Significant factors marked in bold. The significance level was set at *p*-value ≤ 0.05.

**Table 5:** Multivariable logistic regression results for the use of basic water services.

Explanatory Variable	Outcome: Use of Basic Water Service			
		CI 95%		
	OR	Low	Up	*p*-value
Intervention (CBN + WaSH) vs. control (CBN only)	1.00	0.83	1.21	0.968
Oromia vs. Amhara	1.27	0.92	1.75	0.152
SNNPR vs. Amhara	1.11	0.87	1.41	0.417
Tigray vs. Amhara	1.03	0.75	1.40	0.861
Household has electricity * vs. none	2.45	1.90	3.15	**<0.001**
Household head’s highest level of formal education	1.01	0.97	1.07	0.385
MUS of water point vs. no	0.63	0.48	0.84	**0.001**
Water quality is good vs. not good	3.94	3.06	5.08	**<0.001**
Household paid for water in the rainy season vs. no	1.11	0.88	1.40	0.385
Main cause of diarrhoea: drinking unsafe water	1.48	1.20	1.81	**<0.001**

* Electricity used as a proxy variable to wealth in this study. Significant factors marked in bold. The significance level was set at *p*-value ≤ 0.05.

**Table 6:** Bivariate logistic regression results for the use of basic sanitation services.

Outcome is Use of Basic Sanitation Service	Unadjusted Model				Adjusted Model			
		CI 95%				CI 95%		
	OR	Low	Up	*p*-Value	OR	Low	Up	*p*-Value
Explanatory variable								
*Region*				Interv vs. contr	1.24	0.97	1.59	0.084
Oromia vs. Amhara	0.64	0.49	0.83	**0.001**	0.86	0.72	1.03	0.112
SNNPR vs. Amhara	1.15	0.95	1.38	0.150	0.79	0.63	0.98	**0.035**
Tigray vs. Amhara	0.94	0.75	1.18	0.598	1.26	1.03	1.53	**0.023**
Household has electricity * vs. no	1.37	1.14	1.65	**0.001**	1.30	1.04	1.63	**0.022**
Household head’s highest education level	1.02	0.97	1.07	0.352	1.03	0.98	1.08	0.263
Mother’s highest education level	1.02	0.96	1.07	0.514	1.02	0.97	1.08	0.419
*Sanitary hygiene*								
Latrine only cleaned when needed vs. no	1.03	0.84	1.27	0.757	1.08	0.91	1.27	0.365
Latrine has been emptied at least once vs. no	5.16	4.28	6.21	**<0.001**	1.16	0.92	1.46	0.201
Latrine is currently observed to be clean vs. no	0.78	0.60	1.01	0.062	1.16	0.89	1.50	0.265
Latrine observed to be in good condition vs. no	1.08	0.82	1.43	0.576	1.31	1.12	1.53	**0.001**
Household has received training before vs. no	1.51	1.23	1.85	**<0.001**	0.80	0.66	0.96	**0.016**
*Health risk perceptions*								
Latrine benefit: clean compound vs. no	1.67	1.32	2.11	**<0.001**	1.24	1.01	1.53	**0.041**
Latrine benefit: better health vs. no	1.47	1.19	1.82	**<0.001**	1.23	1.03	1.47	**0.020**
Latrine benefit: safer at night vs. no	1.91	1.59	2.28	**<0.001**	1.11	0.95	1.30	0.198
Latrine benefit: better privacy vs. no	2.09	1.78	2.44	**<0.001**	0.80	0.68	0.95	**0.009**
Latrine benefit: better social status vs. no	2.01	1.66	2.44	**<0.001**	0.80	0.68	0.95	**0.009**
Latrine disadvantage: construction costs vs. no	0.41	0.34	0.49	**<0.001**	0.78	0.64	0.96	**0.020**
Latrine disadvantage: maintenance costs vs. no	0.50	0.40	0.61	**<0.001**	1.23	0.85	1.80	0.277
Latrine disadvantage: bad smell/dirt vs. no	1.32	1.08	1.61	**0.006**	1.29	0.96	1.72	0.091
Latrine disadvantage: reason for diarrhoea vs. no	3.20	2.32	4.41	**<0.001**	1.20	1.03	1.41	**0.024**
Diarrhoea reason: dirty space vs. no	1.71	1.45	2.02	**<0.001**	1.16	0.99	1.35	0.065
Diarrhoea reason: handwashing no soap vs. yes	1.33	1.13	1.55	**<0.001**	1.12	0.96	1.31	0.152
Diarrhoea reason: defecate in the open air vs. no	1.26	1.08	1.47	**0.003**	1.10	0.93	1.29	0.269
Diarrhoea reason: drinking unsafe water vs. no	1.39	1.18	1.64	**<0.001**	1.30	1.04	1.63	**0.022**
Diarrhoea reason: Human faeces vs. none	1.00	0.74	1.34	0.999	1.22	0.96	1.54	0.101

* Electricity used as a proxy variable to wealth in this study. ** Significant factors marked in bold. The significance level was set at *p*-value ≤ 0.05.

**Table 7:** Multivariable regression model for the use of basic sanitation services.

Explanatory Variable	Outcome: Use of Basic Sanitation Service			
		CI 95%		
	OR	Low	Up	*p*-Value
Intervention (WaSH = CBN) vs. control (CBN only)	1.41	1.18	1.69	**<0.001**
Oromia vs. Amhara	0.86	0.63	1.16	0.313
SNNPR vs. Amhara	1.58	1.26	1.99	**<0.001**
Tigray vs. Amhara	1.05	0.80	1.38	0.729
Household has electricity * vs. none	1.19	0.95	1.47	0.123
Latrine has been emptied at least once vs. no	6.00	4.86	7.40	**<0.001**
Household has received training before vs. no	1.55	1.22	1.97	**<0.001**
Opinion of main reason for diarrhoea: dirty space vs. no	1.81	1.50	2.19	**<0.001**
Benefit of latrine: better privacy vs. no	2.00	1.67	2.40	**<0.001**
Disadvantage of latrine: maintenance costs vs. no	0.49	0.38	0.63	**<0.001**

* Electricity used as a proxy variable to wealth in this study. Significant factors marked in bold. The significance level was set at *p*-value ≤ 0.05.
